# Impact of Oncotype DX risk categorization and receipt of chemotherapy on survival outcomes among patients with small node-negative HR+ breast cancer

**DOI:** 10.1093/oncolo/oyag276

**Published:** 2026-07-15

**Authors:** Andrea House, Julie A Stephens, Robert Wesolowski, Sachin R Jhawar, Arya Marium Roy, Nerea Lopetegui-Lia, Dionisia Quiroga, Ashley P Davenport, Gilbert Bader, Nicole Williams, Sagar Sardesai, Daniel G Stover, Margaret E Gatti-Mays, Kai C C Johnson

**Affiliations:** College of Medicine, The Ohio State University, Columbus, OH, 43210, United States; College of Medicine, The Ohio State University, Columbus, OH, 43210, United States; Center for Biostatistics, The Ohio State University, Columbus, OH, 43210, United States; Division of Medical Oncology, Department of Internal Medicine, The Ohio State University Comprehensive Cancer Center, Columbus, OH, 43210, United States; Department of Radiation Oncology, The Ohio State University Comprehensive Cancer Center, Columbus, OH, 43210, USA; Division of Medical Oncology, Department of Internal Medicine, The Ohio State University Comprehensive Cancer Center, Columbus, OH, 43210, United States; Division of Medical Oncology, Department of Internal Medicine, The Ohio State University Comprehensive Cancer Center, Columbus, OH, 43210, United States; Division of Medical Oncology, Department of Internal Medicine, The Ohio State University Comprehensive Cancer Center, Columbus, OH, 43210, United States; Division of Medical Oncology, Department of Internal Medicine, The Ohio State University Comprehensive Cancer Center, Columbus, OH, 43210, United States; Division of Medical Oncology, Department of Internal Medicine, The Ohio State University Comprehensive Cancer Center, Columbus, OH, 43210, United States; Division of Medical Oncology, Department of Internal Medicine, The Ohio State University Comprehensive Cancer Center, Columbus, OH, 43210, United States; Division of Medical Oncology, Department of Internal Medicine, The Ohio State University Comprehensive Cancer Center, Columbus, OH, 43210, United States; Division of Medical Oncology, Department of Internal Medicine, The Ohio State University Comprehensive Cancer Center, Columbus, OH, 43210, United States; Division of Medical Oncology, Department of Internal Medicine, The Ohio State University Comprehensive Cancer Center, Columbus, OH, 43210, United States; Division of Medical Oncology, Department of Internal Medicine, The Ohio State University Comprehensive Cancer Center, Columbus, OH, 43210, United States

**Keywords:** Oncotype DX, chemotherapy, T1b, node-negative, hormone receptor-positive breast cancer, national cancer database

## Abstract

**Background:**

The application of genomic assays, such as Oncotype DX, in patients with small (T1mi/a/b) clinically node-negative (N0-N1mi) hormone receptor positive breast cancer (HR+BC) is becoming increasingly popular in real-world clinical practice. Whether these results can be reliably prognostic among this clinically low-risk population and can be used to make decisions on the application of adjuvant chemotherapy remains unknown.

**Patients and methods:**

Data from the National Cancer Database (NCDB) were examined for those diagnosed with small, node-negative HR+BC between 2010 and 2020. Oncotype DX recurrence score (RS) data were classified as low (RS < 11), intermediate (RS = 11-25), or high risk (RS = 26-100). Our primary objective was comparing overall survival (OS) based on chemotherapy receipt for those with high-risk scores. Kaplan-Meier methods and multivariable Cox proportional hazard models were used. Propensity score matching was additionally performed.

**Results:**

Of the 350 911 patients with T1mi/a/b N0/N0(i+)/N1mi HR+BC, 102 357 (29.2%) underwent Oncotype DX testing and had available RS data. Among them, 32 158 (31.4%) had low, 58 891 (57.5%) had intermediate, and 11 308 (11.0%) had high-risk. Chemotherapy receipt was documented in 1.2% (*n *= 402) of low-risk, 6.8% (*n *= 4000) of intermediate-risk, and 66.3% (*n* = 7496) of high-risk patients. Among high-risk patients, those who received chemotherapy had a lower risk of death compared with those in the chemotherapy omission arm, both on univariate (HR, 0.55; 95% CI, 0.52-0.72; *P *< 0.001) and multivariable (HR, 0.61; 95% CI, 0.52-0.72; *P *< 0.001) analyses. This finding remained true following propensity score matching (univariate HR, 0.63; 95% CI, 0.52-0.76; aHR, 0.71; 95% CI, 0.59-0.86; *P *< 0.001 for both). Factors associated with a high RS included higher tumor grade, Black race, and lymphovascular invasion.

**Conclusion:**

Our findings suggest that risk stratification through a genomic assay such as Oncotype DX may be advantageous, even among otherwise clinically low-risk individuals with HR + BC.

Implications for Practice While clinical risk continues to serve as a primary driver for oncology decision making as it pertains to treatment selection for those with small (T1mi/a/b), clinically node-negative, hormone-receptor-positive breast cancer, our study illustrates that genomic risk can still identify potential patients who may benefit from adjuvant chemotherapy. While Oncotype DX testing appears promising, further prospective testing is needed. However, this analysis identified clinicopathologic factors that are highly correlated with high genomic risk classification based on Oncotype DX testing among an otherwise clinically low risk population, including tumor grade, race, and the presence of lymphovascular invasion.

## Introduction

Gene expression profiles have become the standard of care for stratifying clinically low-risk individuals with hormone receptor-positive (HR+) breast cancer (BC) in terms of the necessity for adjuvant chemotherapy in addition to standard endocrine therapy. The Oncotype DX 21-gene assay has become one of the most widely adopted tools used due to its validation in both node-negative and node-positive populations, as seen in the TAILORx and RxPONDER trials, respectively.^1^^,^^2^ In TAILORx, after a 9-year follow-up period, patients with an Oncotype DX recurrence score (RS) ≥26 had an overall survival (OS) of 89.3% when receiving appropriate chemoendocrine therapy versus an OS of 93.7% in those with a RS ≤ 10 on endocrine therapy alone. Those with scores of 11-25 showed similar OS rates whether they received endocrine therapy alone (93.9%) or chemoendocrine therapy (93.8%).

Despite these impressive results for terms of risk stratification, this genomic test has limitations, even with incorporation of the RSClin Tool to account for intrinsic risk factors.^3^ One of the major limitations of Oncotype DX testing is expanding its use in ultra-low-risk individuals, specifically those with small tumor burdens (pT1mi/a/b) and macroscopically node-negative disease on surgical pathology (pN0-pN1mi). Such patients were heavily absent from the landmark trials mentioned previously. Therefore, little is known about whether we should apply Oncotype DX testing within this population, especially given its historically excellent prognosis in the absence of chemotherapy.^4^ However, fear of undertreatment often leads physicians to send the test as a safety measure, providing an opportunity to analyze real-world data on the application and prognostic benefit of Oncotype DX testing among these individuals. In order to answer this research question confidently given the small effect size seen in such a clinically favorable population, we utilized data from the National Cancer Database (NCDB) to perform a large-scale analysis on what the true added benefit of chemotherapy may be among those with clinically low risk disease, which we’re defining as pT1mi/a/b pN0 HR+ BC, with a particular focus on those with genomically high-risk disease (RS 26-100).

## Patients and methods

### Study population

The US NCDB is a national clinical oncology database developed by the American College of Surgeons and the American Cancer Society. It contains deidentified hospital registry data from more than 1500 Commission on Cancer (CoC) accredited facilities and includes approximately 70% of newly diagnosed cancer cases in the United States. The NCDB includes patient demographics, tumor characteristics, primary therapies administered, and some outcomes, including OS. Since all data are deidentified, this study was deemed exempt by The Ohio State University Wexner Medical Center’s Institutional Review Board.

We performed an analysis involving the examination of NCDB survival data for patients diagnosed with small, node-negative HR+ BC between January 1, 2010, and December 31, 2020, who underwent curative breast surgery with available surgical pathologic data in place. For our initial abstract,^5^ we reviewed eligible cases involving estrogen receptor-positive (ER+) and progesterone receptor-positive (PR+) disease; for this updated analysis, we also included those with ER+ and PR-negative (PR-) disease. In our analyses, patients with HER2-positive disease were excluded.

### Study variables

**Figure 1.**
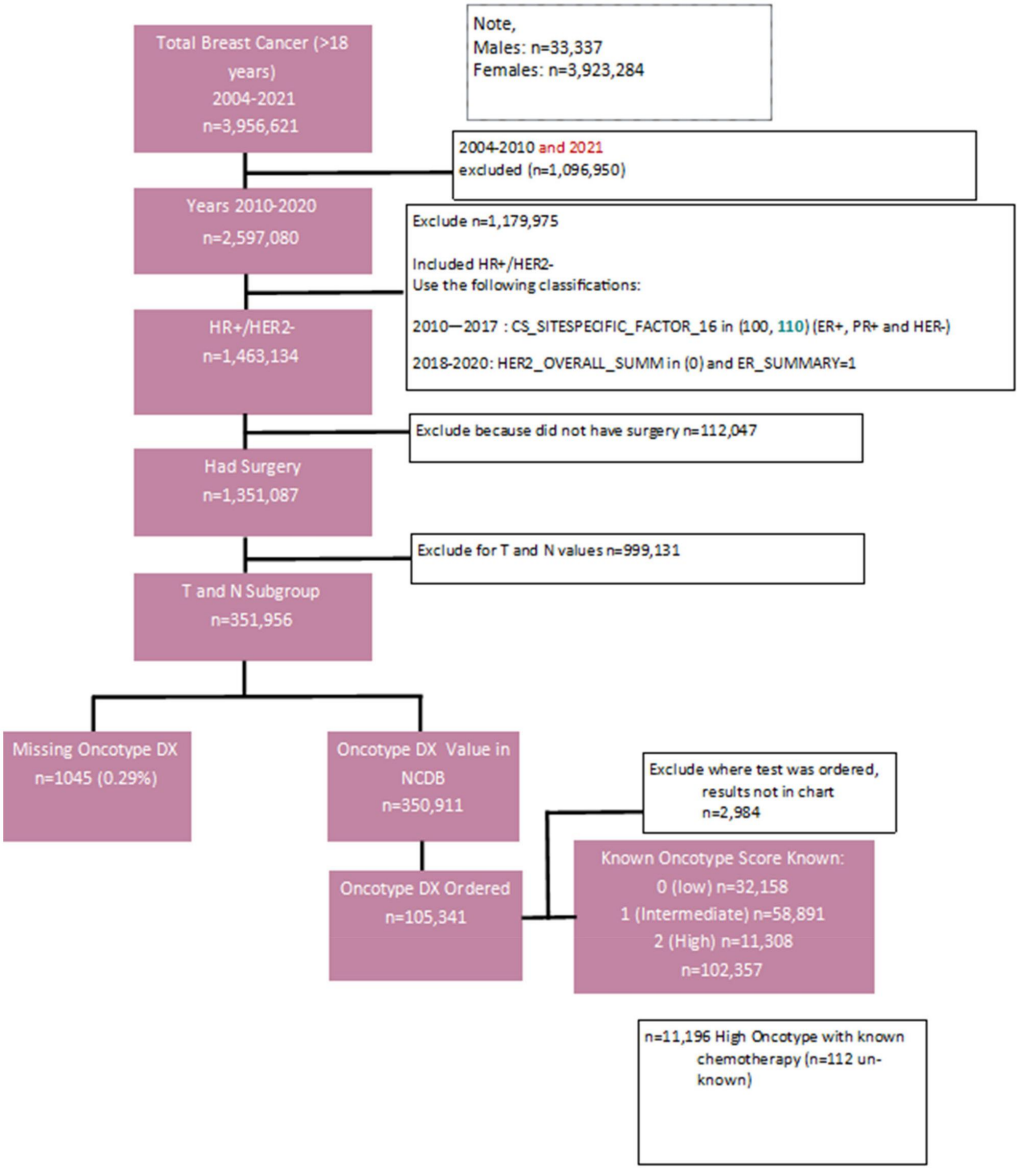
Patient selection algorithm for this retrospective analysis shown via CONSORT diagram.

We examined available Oncotype DX RS data and categorized patients into genomically low- (<11), intermediate- (11-25), and high-risk (26-100) groups. Those for whom the test status was unknown/unavailable were excluded from this study. Additionally, patients who were missing data regarding the administration or omission of chemotherapy were excluded from our analysis. However, missing data as it relates to potentially relevant clinicopathologic and demographic data variables were not exclusionary. Instead, an unknown category was created and included in the modeling performed. An outline of applied eligibility criteria can be found in Figure 1.

Our primary objective was to compare OS among high-risk patients who received chemotherapy versus those for whom chemotherapy was omitted. For our secondary objective, we compared OS between Oncotype DX test recipients and those who did not undergo testing. Additionally, we compared OS between the 3 genomic risk subgroups from within our study population of interest. We defined OS as the time from BC diagnosis to the time of death from any cause or last known follow-up. Common patient demographics and disease characteristics including age (<50/50+), race, ethnicity, T1 substage, tumor grade, micronodal status (pN0/pN(mol−)/pN0(i−) vs pN0(mol+)/pN0(i+)/pN1mi), progesterone receptor status, surgery type, receipt of endocrine therapy, Charlson-Deyo score (0/1+), insurance, facility type, facility location, and year of diagnosis were examined for their relationship with chemotherapy administration and in the models examining OS for the primary variables of interest described above.

### Statistical analysis

Patient demographics, disease characteristics, and outcomes are summarized as frequency (%) for categorical variables and median and interquartile range for continuous variables overall, and by RS group, and by chemotherapy use in the high-RS group. Categorical variables were compared between the groups using χ^2^ tests. Age was compared using a Kruskal–Wallis test. Kaplan–Meier methods were used to estimate OS and generate Kaplan–Meier curves, and the log-rank tests were used to compare survival curves. Univariate Cox proportional hazards were used to evaluate hazard ratios by groups of interest. Multivariable Cox proportional hazard models were used to assess the hazard ratio after adjusting for variables of interest (age, race, Charlson Deyo score, use of endocrine therapy, tumor grade, tumor size, nodal status, progesterone status, lymphovascular invasion, facility type, geographic region, and year of diagnosis). The proportional hazards assumption for the primary outcome was confirmed using the global test as well as graphically. Interaction terms were not included in the model given the analysis is designed to estimate the overall adjusted effect of chemotherapy use rather than evaluate effect modifications. In addition, we did not have prespecified hypotheses for specific interactions and adding multiple interaction terms would increase model complexity and reduce statistical power. Propensity score matching was performed to adjust for baseline differences between treatment groups as a sensitivity analysis. Propensity scores for receipt of chemotherapy among high-risk patients were estimated using logistic regression including age (<50 vs ≥50), Charlson-Deyo comorbidity score (0 vs ≥1), tumor grade (1/2 vs 3), tumor size (T1mi/a vs T1b), nodal status (N0 [N0/N0(i−)/N0(mol−)] vs N+ [N0(i+)/N0(mol+)/N1mi]), endocrine therapy use, and progesterone receptor status. One-to-one nearest-neighbor matching with a caliper of 0.2 standard deviations of the logit of the propensity score and restriction to the region of common support was performed. Covariate balance was assessed using standardized mean differences, with all post-matching values <0.10 indicating adequate balance. Overall survival in the matched cohort was analyzed using Cox proportional hazards models with robust standard errors clustered on matched pairs and adjustment for covariates with residual imbalance. Analyses were conducted using either SAS 9.4 (SAS Institute Inc., Cary, NC) or Stata 16.1 (StataCorp, College Station, TX). An alpha level of 0.05 was used to determine statistical significance.

## Results

Of the 350 911 patients within the NCDB whose cases were documented as clinically node-negative T1mi/a/b HR+ BC, a total of 102 452 underwent Oncotype DX testing; of those, only 11 308 (11.1%) were determined to have high Oncotype DX RSs. Among these high-risk individuals, 7496 received chemotherapy, whereas the remaining 3700 patients did not. A summary of baseline characteristics by chemotherapy can be found in Table 1.

**Table 1.** Summary of baseline characteristics among high genomic risk patients based on chemotherapy receipt.

	Chemotherapy (*n *= 7496)	No chemotherapy (*n *= 3700)	**Total (*N *= 11 196)** ^a^	*P*-value
**Age**	<50: 1510 (20.1%) ≥50: 5986 (79.9%)	<50: 378 (10.2%) ≥50: 3322 (89.8%)	<50: 1888 (16.9%) ≥50: 9308 (83.1%)	<.0001
**Mean (SD)**	58.1 (10.00)	63.2 (10.05)	59.8 (10.30)	
**Median [Min, Max]**	59.0 [23.0-90.0]	64.0 [22.0-89.0]	61.0 [22.0-90.0]	
**Race**				.6837
** Black**	781 (9.9%)	366 (9.9%)	1147 (10.2%)	
** White/other**	6649 (88.7%)	3302 (89.2%)	9951 (88.9%)	
**Ethnicity**				.3958
** Hispanic or Latino**	349 (4.7%)	152 (4.1%)	501 (4.5%)	
** Non-Hispanic**	6966 (92.9%)	3462 (93.6%)	10 428 (93.1%)	
**Charlson-Deyo Score**				.0041
** 0**	6390 (85.2%)	3077 (83.2%)	9467 (84.6%)	
** ≥1**	1106 (14.8%)	623 (16.8%)	1729 (15.4%)	
**T stage**				<.0001
** T1a**	914 (12.2%)	584 (15.8%)	1498 (13.4%)	
** T1b**	6547 (87.3%)	3100 (83.8%)	9647 (86.2%)	
** T1mic**	35 (0.5%)	16 (0.4%)	51 (0.5%)	
**Tumor grade**				<.0001
** Low/intermediate**	4196 (78.4%)	2767 (88.7%)	6963 (82.2%)	
** High**	984 (18.4%)	260 (8.3%)	1244 (14.7%)	
**Nodal status**				.0124
** N0/N0(i**−**)/N0(mol**−**)**	7040 (93.9%)	3518 (95.1%)	10 558 (94.3%)	
** N0(i+)/N0(mol+)/N1mi**	456 (6.1%)	182 (4.9%)	638 (5.7%)	
**Surgery type**				.0183
** Lumpectomy**	3652 (48.7%)	1893 (51.2%)	5545 (49.5%)	
** Mastectomy**	3844 (51.3%)	1806 (48.8%)	5650 (50.5%)	
**Progesterone receptor status**				<.0001
** Positive**	4974 (66.4%)	2752 (74.4%)	7762 (69.0%)	
** Negative**	2519 (33.6%)	945 (25.5%)	3464 (30.9%)	
**Endocrine therapy receipt**				<.0001
** Yes**	6525 (87.0%)	3130 (84.6%)	9655 (86.2%)	
** No**	735 (9.8%)	486 (13.1%)	1221 (10.9%)	
**Facility type**				.0045
** Community Cancer Program**	460 (6.4%)	264 (7.3%)	724 (6.7%)	
** Comprehensive Community Cancer Program**	2843 (39.5%)	1519 (41.7%)	4362 (40.2%)	
** Academic/Research Program**	2374 (33.0%)	1087 (29.9%)	3461 (31.9%)	
** Integrated Network Cancer Program**	1522 (21.1%)	771 (21.2%)	2293 (21.2%)	
**Region**				.0004
** New England**	460 (6.4%)	227 (6.2%)	687 (6.3%)	
** Middle Atlantic**	1453 (20.2%)	681 (18.7%)	2134 (19.7%)	
** South Atlantic**	1553 (21.6%)	904 (24.8%)	2457 (22.7%)	
** East North Central**	1332 (18.5%)	605 (16.6%)	1937 (17.9%)	
** East South Central**	364 (5.1%)	196 (5.4%)	560 (5.2%)	
** West North Central**	628 (8.7%)	267 (7.3%)	895 (8.3%)	
** West South Central**	394 (5.5%)	212 (5.8%)	606 (5.6%)	
** Mountain**	335 (4.7%)	167 (4.6%)	502 (4.6%)	
** Pacific**	680 (9.4%)	382 (10.5%)	1062 (9.8%)	
**Year of diagnosis**				.0019
** 2010**	378 (5.0%)	192 (5.2%)	570 (5.1%)	
** 2011**	469 (6.3%)	236 (6.4%)	705 (6.3%)	
** 2012**	489 (6.5%)	224 (6.1%)	713 (6.4%)	
** 2013**	616 (8.2%)	288 (7.8%)	904 (8.1%)	
** 2014**	681 (9.1%)	328 (8.9%)	1009 (9.0%)	
** 2015**	722 (9.6%)	381 (10.3%)	1103 (9.9%)	
** 2016**	830 (11.1%)	459 (12.4%)	1289 (11.5%)	
** 2017**	859 (11.5%)	481 (13.0%)	1340 (12.0%)	
** 2018**	795 (10.6%)	423 (11.4%)	1218 (10.9%)	
** 2019**	893 (11.9%)	364 (9.8%)	1257 (11.2%)	
** 2020**	764 (10.2%)	324 (8.8%)	1088 (9.7%)	

a Data for 107 patients with unknown chemotherapy receipt status were excluded from analysis.

**Figure 2.**
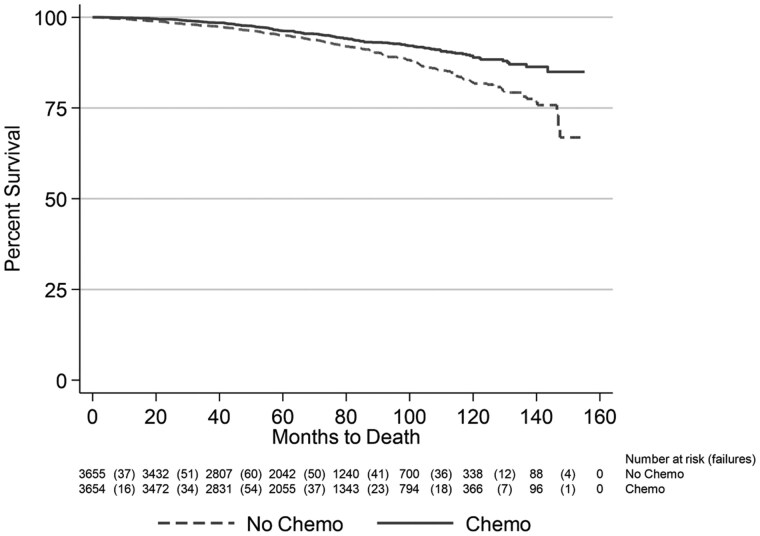
Univariate analysis of OS based on receipt of adjuvant chemotherapy for patients with high-risk Oncotype DX recurrence scores following propensity score matching.

For our primary objective, among high-risk patients, those who received chemotherapy had a lower risk of death than those in the chemotherapy omission arm, (HR, 0.55; 95% CI, 0.52-0.72; *P *< 0.001; Figure S1). Specifically, the 5- and 10-year OS rates for chemotherapy recipients with high-risk disease were 96.7% (96.2-97.2%) and 90.4% (89.2-91.5%); for the omission group, it was 94.9% (94.1-95.7%) and 81.9% (79.3-84.3%), respectively. The survival benefit remained significant after controlling for age, race, co-morbidity score, tumor size, nodal status, tumor grade, lymphovascular invasion, progesterone receptor status, receipt of endocrine therapy, facility type, geographic region, and year of diagnosis (HR, 0.61; 95% CI, 0.52-0.72; *P *< 0.001) (Table 2). Lastly, we performed propensity score matching, resulting in 3655 matched patients in each arm for our genomically high-risk patients. We found that following matching, survival benefit remained significant with chemotherapy receipt compared with omission (univariate HR, 0.63; 95% CI, 0.52-0.76; aHR, 0.71; 95% CI, 0.59-0.86; *P *< 0.001 for both) (Figure 2).

**Table 2.** Univariate and multivariable Cox proportional hazards models.^a^

	OS	5-year OS	10-year OS
**Univariate model**			
**Chemotherapy vs chemotherapy omission (reference)**	HR 0.55 95% CI, 0.52-0.72 *P *< 0.001^b^	Chemotherapy: 97.0% (96.5-97.4%) Omission: 94.7% (93.9-95.4%)	Chemotherapy: 90.1% (89.5-91.9%) Omission: 82.2% (79.8-84.4%)
**Oncotype DX testing vs omission of testing (reference)**	HR 0.45 95% CI, 0.44-0.47 *P *< 0.001^b^	Testing: 97.2% (97.0-97.3%) No testing: 93.6% (93.5-93.7%)	Testing: 90.5% (90.2-90.8%) No testing: 80.7% (80.4-80.9%)
**Oncotype DX high vs intermediate/low risk (reference)**	HR 1.36 95% CI, 1.26-1.48 *P *< 0.001^b^	Low/Intermediate risk: 97.3% (97.2-97.4%) High risk: 96.1% (95.7-96.5%)	Low/Intermediate risk: 90.0% (90.5-91.2%) High risk: 87.7% (86.5-88.8%)
**Multivariable model** ^a^			
**Chemotherapy vs chemotherapy omission (reference)**	HR 0.61 95% CI, 0.52-0.72 *P *< 0.001^b^	N/A	
**Oncotype DX testing vs omission of testing (reference)**	HR 0.58 95% CI 0.56-0.60 *P *< 0.001^b^		
**Oncotype DX high vs intermediate/low risk (reference)**	HR 1.22 95% CI, 1.12-1.34 *P *< 0.001^b^		

Abbreviations: CI, confidence interval; HR, hazard ratio; OS, overall survival.

a Adjusting for age, race, charlson-deyo score, endocrine therapy, tumor grade, tumor size, nodal status, lymphovascular invasion, progesterone receptor status, facility type, geographic region, & year of diagnosis.

b Denotes statistical significance between comparator groups.

Oncotype DX testing was associated with a substantially lower hazard of OS, about a 45% reduction in risk of death, compared with not having the test (HR, 0.45; 95% CI, 0.44-0.47; *P *< 0.001; Figure S2) and multivariable modeling (HR, 0.58; 95% CI, 0.56-0.60; *P *< 0.001). Specifically, the 5-year overall survival for the Oncotype Testing Group was 97.2% vs 93.6% in the omission arm. The OS was also significantly different between the Oncotype DX risk categories (low, intermediate, and high), with patients in the low- and intermediate-risk groups demonstrating superior survival compared with those in the high-risk group (*P *< 0.001; Figure S3). The corresponding 5-year OS rates were 97.0% for low-risk patients, 97.5% for intermediate-risk patients, and 96.1% for high-risk patients. Differences in demographic characteristics between Oncotype DX tested and untested patients are shown in Table S1. This included a higher percentage of patients who received endocrine therapy in the Oncotype DX tested group versus the omission group. The Kaplan-Meier curves for OS by both Oncotype DX risk grouping and chemotherapy receipt are shown in Figure S4.

For our exploratory analysis, we examined what relevant clinicopathologic features contributed to Oncotype DX risk categorizations. We found that those with high-risk disease were more commonly found to have a high tumor grade (35.5% for high risk, 7.1% for intermediate risk, and 3.8% for low risk); lymphovascular invasion (6.8% high risk, 4.4% intermediate risk, 4.2% low risk); and racially identify as African American (10.2% high risk, 6.8% intermediate risk, 6.7% low risk). These findings are shown in Table S2. Additionally, we explored OS by Oncotype DX risk category/chemotherapy, as well as OS stratified by nodal statuses, categorized as N0 (N0/)/N0(mol−) vs N+ (N0(i+)/N0(mol+)/N1mi), and T1 substages (T1mi, T1a, T1b). For those patients with T1mi N0 or T1mi N+ HR+ BC, no difference in OS was noted between low-, intermediate-, and high-risk patients. For patients with T1a N0 disease, a measurable improvement in OS was noted among low- and intermediate-risk patients (*P *= 0.025), but this was not seen among T1aN+ patients based on Oncotype DX risk categories (*P *= 0.737). Lastly, among patients with T1bN0 disease, the strongest difference in OS was seen between risk categories (*P *< 0.001), and this association remained true among patients with T1bN+ disease (*P *= 0.002). These findings are illustrated in Table S3, and the 5-year OSs for these groups are shown in Table S4, as well as an age-specific breakdown in Table S5. The Kaplan–Meier curves are provided with Figures S5-S7, for T1mi, T1a, and T1b subgroups, respectively.

## Discussion

This study demonstrates that among patients with small (T1mi/a/b), clinically node-negative (pN0-pN1mi), early-stage, HR+, clinically low-risk BC but genomically high-risk disease based on Oncotype DX RS testing, a clinically and statistically meaningful improvement in OS was seen when chemotherapy is given. However, this study population predominantly involved pT1b disease; while the risk of death was nearly twice as high in patients who did not receive chemotherapy, the absolute difference in 5-year mortality was modest. The number needed to treat was roughly 44 patients to prevent 1 death at 5 years, so while benefit was seen, further treatment personalization tools need to be developed and applied. In terms of the prognostic value of Oncotype DX genomic risk categories among this clinically low-risk population, when comparing patients with low/intermediate-risk scores to those with high-risk scores, patients in the high-risk group had worsened OS. Lastly, receipt of Oncotype DX testing alone was also associated with an increase in OS, which is in part due to the escalation of adjuvant therapy when a high Oncotype DX RS is discovered. This is exploratory in nature given issues with selection bias which cannot be completely accounted for retrospectively.

While our findings appear consistent with data reported within prospective studies, including TAILORx, these clinically low-risk patients were often excluded or underrepresented in such studies. When reviewing available retrospective data on this pT1mi/a/b pN0/N1mi population, we found that data were similarly limited. An institutional retrospective analysis^6^ found that the performance of Oncotype DX testing was associated with an improvement in OS on univariate analysis (93.2% vs 83.2%, *P *< 0.01). This difference in OS became non-significant when adjusted for other variables in a multivariable model (*P *= 0.73), though the disease-free survival (DFS) benefit remained significant (*P *< 0.01), particularly among pT1b patients (*P *< 0.01) as compared with pT1a patients (*P *= 0.26). Additionally, OS was significantly improved between low-risk and high-risk patients (*P *< 0.01), with an absolute difference in OS of 44 months between groups. Lastly, the application of Oncotype DX testing among this population ranged from 31.6-56.2% of patients, with only 4.4% demonstrating a high-risk RS (3.6% of pT1a patients and 4.6% of pT1b patients). Of note, the cutoff for high risk used for this study was an RS > 30, not the more traditional RS > 26 cutoff used today. A separate institutional retrospective analysis^7^ reported univariate results showing that, for those with pT1a/pT1b pN0 HR+ BC, distant recurrence rates were associated with a higher RS (*P *= 0.024), receipt of adjuvant chemotherapy (*P *= 0.002), and grade 3 disease (*P *= 0.012). For DFS however, only grade 3 disease (*P *< 0.001) and receipt of adjuvant chemotherapy (*P *= 0.001) were associated with worsened DFS on univariate analysis, and this remained the case in the multivariable model. There have been studies, however, demonstrating limited chemotherapy benefit among this population. A small retrospective observational cohort analysis by Katz D et al.^8^ examining invasive DFS among women with high RS T1a/b N0 HR+BC reported no meaningful difference in invasive DFS event rates among the 74 propensity score matched patients in the chemotherapy administration versus chemotherapy omission cohorts (*P *= 0.39). However, the event rate was low, and interestingly, the median time to captured invasive DFS events was 12 years, though this was a 10-year follow-up study. Similarly, a small dedicated exploratory analysis of MINDACT data by Hilbers et al.^9^ reported that for patients with pT1a-b pN0 HR+BC that are genomically high risk based on MammaPrint testing, no meaningful difference in 8-year DFS rate was seen (HR, 1.19; 95% CI, 0.43-3.27) between chemotherapy omission (*n* = 54) versus chemotherapy administration (*n* = 42). That said, event rates were low in each arm, chemotherapy arms were assigned by intention-to-treat as opposed to receipt of chemotherapy, and these exploratory cohorts were not balanced based on administration of endocrine therapy. Interestingly, DFS benefit appeared improved among those below the age of 50, but this study was too underpowered for subgroup analysis.

The question of whether clinicopathologic features alone can predict Oncotype DX RS among this population has been investigated previously. One study by Markopoulos and colleagues, involving Oncotype DX testing data from 5 separate Greek institutes showed that among primarily small (pT1/T2), macroscopically node-negative (pN0/pN1mi), HR+ BC patients, the individual presence of additional unfavorable characteristics, such as micrometastases, high tumor grade, and larger tumor size (pT2) had no impact on RS as a continuous variable.^10^ However, Ki-67 as an individual factor was associated with RS, both univariately (*P *< 0.001) and after adjusting for other variables in a multivariable model (*P *= 0.009). This held true when looking at the relationship between categorical Ki-67 and RS risk groups (*P *= 0.001), with a high RS (cutoff > 30) being predictive of high Ki-67 (≥ 20%), though the opposite was not true. A separate, multi-institutional retrospective study^11^ showed conflicting results, with their multivariable model demonstrating that tumor grade showed a significant positive association with RS, whether they used old (high RS > 30) or new (high RS > 26) RS cutoffs.

In terms of our own findings relating to predictors of RS categorizations, we found that a high tumor grade, presence of lymphovascular invasion, and racially identifying as Black were all associated with a high Oncotype DX RS. In addition, we reviewed data pertaining to specific tumor size and microscopic nodal subgroups (pT1mi pN0/pT1mi pN1mi+/pT1a pN0/pT1a pN1mi+/pT1b pN0/pT1b pN1mi+) and found that those with genomically high-risk pT1b disease, regardless of micrometastatic nodal status, had a clear improvement in OS. These cumulative findings underscore the importance of exercising caution when using clinical features alone to predict recurrence risk.

The strengths of our study include sample size and multi-institutional status, which helped improve the confidence and generalizability of our findings. In addition, we had access not only to the risk group categorizations, but also to the Oncotype DX RS values themselves, allowing us to use an updated cutoff for the risk groupings. In terms of limitations, the retrospective nature of this study comes with inherent selection bias issues, even when performing multivariable analyses and propensity score matching to overcome this. Furthermore, although we examined the benefit of chemotherapy, the details regarding doses administered, duration of therapy, and specific chemotherapy agents applied are unknown. This is similarly true for endocrine therapy, which is particularly an issue given the known survival impacts that duration of therapy, type of endocrine therapy agent applied, and use of ovarian suppression among pre-menopausal patients can have for patients with HR+ BC. The menopausal status of patients was also unknown, with an age cutoff of 50 years being applied as an approximation only. Details relating to locoregional therapy, including surgery and radiation therapy, were also limited given the scope of this analysis. Lastly, the use of OS as our primary outcome as opposed to a more recurrence-focused endpoint, such as invasive recurrence-free survival, limits our ability to detect smaller impacts on treatment benefit; however, OS remains a clinically meaningful endpoint to examine, even if event rates are low overall.

This study provides important data to consider when applying Oncotype DX testing among clinically low-risk patients. The association between chemotherapy receipt and increased overall survival among those found to be genomically high risk is clinically and statistically significant but was most clinically meaningful among those with tumors measuring 5-10 mm in size on surgical pathology (pT1b), including those with negative and microscopic nodal involvement. These study findings further support the concept of treatment personalization based on tumor biology, with the hope that future stratification tools may aid in selecting those who may benefit from adjuvant chemotherapy.

## Supplementary Material

oyag276_Supplementary_Data

## Data Availability

The data generated in this study are available upon request from the corresponding author.

## References

[1] Sparano JA , Gray RJ, Makower DF, et al Adjuvant chemotherapy guided by a 21-gene expression assay in breast cancer. N Engl J Med. 2018;379:111-121. 10.1056/NEJMoa1804710 29860917 PMC6172658

[2] Kalinsky K , Barlow WE, Gralow JR, et al 21-Gene assay to inform chemotherapy benefit in Node-Positive breast cancer. N Engl J Med. 2021;385:2336-2347. 10.1056/NEJMoa2108873 34914339 PMC9096864

[3] Sparano JA , Crager MR, Tang G, Gray RJ, Stemmer SM, Shak S. Development and validation of a tool integrating the 21-gene recurrence score and clinical-pathological features to individualize prognosis and prediction of chemotherapy benefit in early breast cancer. J Clin Oncol. 2021;39:557-564. 10.1200/JCO.20.03007 33306425 PMC8078482

[4] Early Breast Cancer Trialists’ Collaborative Group (EBCTCG). Effects of chemotherapy and hormonal therapy for early breast cancer on recurrence and 15-year survival: an overview of the randomised trials. Lancet. 2005;365:1687-1717. 15894097 10.1016/S0140-6736(05)66544-0

[5] Johnson K , Stephens JA, Sandoval B, et al Abstract P1-11-06: impact of OncotypeDx risk categorization & receipt of chemotherapy on survival outcomes among patients with small (T1mi/a/b) node-negative (N0/N0(i+)/N1mi) hormone receptor positive (HR+) breast cancer. Clinical Cancer Research. 2025;31:1-11. 10.1158/1557-3265.SABCS24-P1-11-06

[6] Pomponio M , Keele L, Hilt E, et al Impact of 21-gene expression assay on clinical outcomes in node-negative ≤ T1b breast cancer. Ann Surg Oncol. 2020;27:1671-1678. 10.1245/s10434-019-08028-w 31686348

[7] Nguyen TTA , Postlewait LM, Zhang C, et al Utility of Oncotype DX score in clinical management for T1 estrogen receptor positive, HER2 negative, and lymph node negative breast cancer. Breast Cancer Res Treat. 2022;192:509-516. 10.1007/s10549-022-06530-6 35084624

[8] Katz D , Feldhamer I, Wolff-Sagy Y, Goldvaser H, Hammerman A, Goldstein DA. Adjuvant chemotherapy in T1a/bN0 breast cancer with a high 21-gene recurrence score (> 25): a 10-year follow-up in a real-world cohort. Breast Cancer. 2025;32:286-291. 10.1007/s12282-024-01652-9 39602054 PMC11842534

[9] Hilbers FS , Poncet C, Tryfonidis K, et al Benefit of systemic therapy in MINDACT patients with small, ER-positive, HER2-negative breast cancers. NPJ Breast Cancer. 2024;10:97. 10.1038/s41523-024-00670-2 39488549 PMC11531586

[10] Markopoulos C , Xepapadakis G, Venizelos V, et al Clinical experience of using Oncotype DX as an additional treatment decision tool in early breast cancer— a retrospective analysis from 5 Greek institutions. Eur J Surg Oncol. 2012;38:413-419. 10.1016/j.ejso.2012.02.183 22425282

[11] Singh K , He X, Kalife ET, Ehdaivand S, Wang Y, Sung CJ. Relationship of histologic grade and histologic subtype with Oncotype Dx recurrence score; retrospective review of 863 breast cancer oncotype Dx results. Breast Cancer Res Treat. 2018;168:29-34. 10.1007/s10549-017-4619-4 29230662

