# Supplementary material for: Impact of Oncotype DX risk categorization and receipt of chemotherapy on survival outcomes among patients with small node-negative HR+ breast cancer
Source: Oncologist. 2026 Jul 15;31(8):oyag276. doi: 10.1093/oncolo/oyag276 (PMC13407421; doi:10.1093/oncolo/oyag276)
Supplement: oyag276_Supplementary_Data [file oyag276_supplementary_data.zip › Supplementary Appendix - Final.docx]

**Supplemental Tables**

**Table S1:** Summary of baseline characteristics overall and based on Oncotype DX testing

|  | **Oncotype DX omitted** (*n*=245570) | **Oncotype DX performed** (*n*=105341) | **Total** (*n*=350911) | ***p*-value** |
| --- | --- | --- | --- | --- |
| Age | <50: 27248 (11.1%)  ≥50: 218322 (88.9%) | <50: 19769 (18.8%)  ≥50: 85572 (81.2%) | <50: 47017 (13.4%)  ≥50: 303894 (86.6%) | <0.0001 |
| Mean (SD) | 64.5 (11.26) | 59.5 (10.20) | 63.0 (11.19) |  |
| Median [Min, Max] | 66.0 [19.0-90.0] | 60.0 [19.0-90.0] | 64.0 [19.0-90.0] |  |
| Race |  |  |  | <0.0001 |
| Black | 19577 (8.0%) | 7565 (7.2%) | 27142 (7.7%) |  |
| White/Other | 224041 (91.2%) | 96862 (92.0%) | 320903 (91.4%) |  |
| Ethnicity |  |  |  | 0.0005 |
| Hispanic or Latino | 11614 (4.7%) | 4686 (4.4%) | 16300 (4.6%) |  |
| Non-Hispanic | 227799 (92.8%) | 98089 (93.1%) | 325888 (92.9%) |  |
| Charlson-Deyo Score |  |  |  | <0.0001 |
| 0 | 202701 (82.5%) | 89741 (85.2%) | 292442 (83.3%) |  |
| ≥1 | 42869 (17.5%) | 15600 (14.8%) | 58469 (16.7%) |  |
| T stage |  |  |  | <0.0001 |
| T1a | 86747 (35.3%) | 12710 (12.1%) | 99457 (28.3%) |  |
| T1b | 150036 (61.1%) | 92277 (87.6%) | 242313 (69.1%) |  |
| T1mic | 8787 (3.6%) | 354 (0.3%) | 9141 (2.6%) |  |
| Tumor grade |  |  |  | <0.0001 |
| Low/intermediate | 218280 (88.9%) | 92928 (88.2%) | 311208 (88.7%) |  |
| High | 15346 (6.2%) | 9634 (9.1%) | 24980 (7.1%) |  |
| Nodal status |  |  |  | <0.0001 |
| N0/N0(i-)/N0(mol-) | 237553 (96.7%) | 98683 (93.7%) | 336236 (95.8%) |  |
| N0(i+)/N0(mol+)/N1mi | 8017 (3.3%) | 6658 (6.3%) | 14675 (4.2%) |  |
| Progesterone Receptor Status |  |  |  | <0.0001 |
| Positive | 220487 (89.8%) | 95560 (90.7%) | 316047 (90.1%) |  |
| Negative | 24951 (10.2%) | 9730 (9.2%) | 34681 (9.9%) |  |
| Surgery type |  |  |  | <0.0001 |
| Lumpectomy | 123385 (50.2%) | 53780 (51.1%) | 177165 (50.5%) |  |
| Mastectomy | 122091 (49.7%) | 51536 (48.9%) | 173627 (49.5%) |  |
| Endocrine therapy receipt |  |  |  | <0.0001 |
| Yes | 186380 (75.9%) | 94352 (89.6%) | 280732 (80.0%) |  |
| No | 51355 (20.9%) | 8634 (8.2%) | 59989 (17.1%) |  |
| Facility Type |  |  |  | <0.0001 |
| Community Cancer Program | 16688 (6.9%) | 7230 (7.0%) | 23918 (6.9%) |  |
| Comprehensive Community Cancer Program | 101989 (42.1%) | 41852 (40.6%) | 143841 (41.7%) |  |
| Academic/Research Program | 70547 (29.1%) | 31607 (30.7%) | 102154 (29.6%) |  |
| Integrated Network Cancer Program | 52807 (21.8%) | 22293 (21.6%) | 75100 (21.8%) |  |
| Facility Location |  |  |  | <0.0001 |
| New England | 17722 (7.3%) | 6369 (6.2%) | 24091 (7.0%) |  |
| Middle Atlantic | 35707 (14.8%) | 22376 (21.7%) | 58083 (16.8%) |  |
| South Atlantic | 53547 (22.1%) | 22765 (22.1%) | 76312 (22.1%) |  |
| East North Central | 40011 (16.5%) | 18322 (17.8%) | 58333 (16.9%) |  |
| East South Central | 14176 (5.9%) | 5016 (4.9%) | 19192 (5.6%) |  |
| West North Central | 18923 (7.8%) | 8308 (8.1%) | 27231 (7.9%) |  |
| West South Central | 18549 (7.7%) | 5143 (5.0%) | 23692 (6.9%) |  |
| Mountain | 11248 (4.6%) | 5098 (5.0%) | 16346 (4.7%) |  |
| Pacific | 32148 (13.3%) | 9585 (9.3%) | 41733 (12.1%) |  |
| Year of Diagnosis |  |  |  | <0.0001 |
| 2010 | 18933 (7.7%) | 4857 (4.6%) | 23790 (6.8%) |  |
| 2011 | 20464 (8.3%) | 6345 (6.0%) | 26809 (7.6%) |  |
| 2012 | 21069 (8.6%) | 7130 (6.8%) | 28199 (8.0%) |  |
| 2013 | 22205 (9.0%) | 8363 (7.9%) | 30568 (8.7%) |  |
| 2014 | 22589 (9.2%) | 8996 (8.5%) | 31585 (9.0%) |  |
| 2015 | 22992 (9.4%) | 9874 (9.4%) | 32866 (9.4%) |  |
| 2016 | 23472 (9.6%) | 11035 (10.5%) | 34507 (9.8%) |  |
| 2017 | 24151 (9.8%) | 11747 (11.2%) | 35898 (10.2%) |  |
| 2018 | 23755 (9.7%) | 11924 (11.3%) | 35679 (10.2%) |  |
| 2019 | 24757 (10.1%) | 13264 (12.6%) | 38021 (10.8%) |  |
| 2020 | 21183 (8.6%) | 11806 (11.2%) | 32989 (9.4%) |  |

Abbreviations: CI = confidence interval; OS = overall survival

**Table S2:** Summary of baseline characteristics across Oncotype DX genomic risk categorizations

| **Oncotype Recurrence Risk Category** | **Low**  (*n*=32158) | **Intermediate** (*n*=58891) | **High**  (*n*=11308) | **Total** (*N*=102357) | ***p*-value** |
| --- | --- | --- | --- | --- | --- |
| Age | <50: 5459 (17.0%)  ≥50: 26699 (83.0%) | <50: 11834 (20.1%)  ≥50: 47057 (79.9%) | <50: 1906 (16.9%)  ≥50: 9402 (83.1%) | <50: 19199 (18.8%)  ≥50: 83158 (81.2%) | <0.0001 |
| Mean (SD) | 60.4 (10.10) | 59.0 (10.17) | 59.8 (10.30) | 59.5 (10.18) |  |
| Median [Min, Max] | 62.0 [22.0-90.0] | 60.0 [19.0-90.0] | 61.0 [22.0-90.0] | 60.0 [19.0-90.0] |  |
| Race |  |  |  |  | <0.0001 |
| Black | 2418 (6.7%) | 4031 (6.8%) | 1161 (10.3%) | 7340 (7.2%) |  |
| White/Other | 29737 (92.5%) | 54356 (92.3%) | 10047 (88.8%) | 94140 (92.0%) |  |
| Ethnicity |  |  |  |  | 0.4267 |
| Hispanic or Latino | 1467 (4.6%) | 2560 (4.3%) | 511 (4.5%) | 4538 (4.4%) |  |
| Non-Hispanic | 29887 (92.9%) | 54928 (93.9%) | 10526 (93.1%) | 95341 (93.1%) |  |
| T stage |  |  |  |  | <0.0001 |
| T1a | 4394 (13.7%) | 6167 (10.5%) | 1517 (13.4%) | 12078 (11.8%) |  |
| T1b | 27632 (85.9%) | 52601 (89.3%) | 9739 (86.1%) | 89972 (87.9%) |  |
| T1mic | 132 (0.4%) | 123 (0.2%) | 52 (0.4%) | 307 (0.3%) |  |
| Microscopic Nodal Status |  |  |  |  | <0.0001 |
| N0 | 29985 (93.2%) | 55254 (93.8%) | 10662 (94.3%) | 95901 (93.7%) |  |
| N+ | 2173 (6.8%) | 3637 (6.2%) | 646 (5.7%) | 6456 (6.3%) |  |
| Tumor grade |  |  |  |  | <0.0001 |
| Low/ intermediate | 30028 (93.4%) | 53257 (90.4%) | 7030 (62.2%) | 90315 (88.2%) |  |
| High | 1218 (3.6%) | 4164 (7.1%) | 4011 (35.5%) | 9393 (9.2%) |  |
| Lymphovascular Invasion |  |  |  |  | <0.0001 |
| Present | 1335 (4.2%) | 2580 (4.4%) | 767 (6.8%) | 4682 (4.6%) |  |
| Not Present | 27530 (85.6%) | 50508 (85.8%) | 9344 (82.6%) | 87382 (85.4%) |  |
| Unknown | 3293 (10.2%) | 5803 (9.8%) | 1197 (10.5%) | 10293 (10.0%) |  |
| Surgery type |  |  |  |  | <0.0001 |
| Lumpectomy | 15648 (48.7%) | 31145 (52.9%) | 5582 (49.4%) | 52375 (51.2%) |  |
| Mastectomy | 16500 (51.3%) | 27735 (47.1%) | 5725 (50.6%) | 49960 (48.8%) |  |
| Progesterone Receptor Status |  |  |  |  | <0.0001 |
| Positive | 31312 (97.4%) | 53786 (91.3%) | 7788 (68.9%) | 92886 (90.7%) |  |
| Negative | 828 (97.4%) | 5080 (8.6%) | 3514 (31.1%) | 9422 (9.2%) |  |
| Endocrine Therapy Use |  |  |  |  | <0.0001 |
| Yes | 28630 (89.0%) | 53667 (91.1%) | 9681 (85.6%) | 91978 (89.9%) |  |
| No | 2874 (8.9%) | 4076 (6.9%) | 1240 (11.0%) | 8190 (8.0%) |  |
| Charlson-Deyo Score |  |  |  |  | <0.0001 |
| 0 | 27112 (84.3%) | 50467 (85.7%) | 9565 (84.6%) | 87144 (85.1%) |  |
| ≥1 | 5046 (15.7%) | 8424 (14.3%) | 1743 (15.4%) | 15213 (14.9%) |  |
| Facility Type |  |  |  |  | <0.0001 |
| Community Cancer Program | 2400 (7.6%) | 3857 (6.7%) | 729 (6.7%) | 6986 (7.0%) |  |
| Comprehensive Community Cancer Program | 13067 (41.3%) | 23235 (40.4%) | 4398 (40.2%) | 40701 (40.7%) |  |
| Academic/Research Program | 9313 (29.4%) | 18005 (31.3%) | 3501 (32.0%) | 30819 (30.8%) |  |
| Integrated Network Cancer Program | 6858 (21.7%) | 12401 (21.6%) | 2318 (21.2%) | 21577 (21.6%) |  |
| Facility Location |  |  |  |  | <0.0001 |
| New England | 1840 (5.8%) | 3662 (6.4%) | 693 (6.3%) | 6195 (6.2%) |  |
| Middle Atlantic | 6872 (21.7%) | 12748 (22.2%) | 2160 (19.7%) | 21780 (21.8%) |  |
| South Atlantic | 7240 (22.9%) | 12370 (21.5%) | 2477 (22.6%) | 22087 (22.1%) |  |
| East North Central | 5702 (18.0%) | 10141 (17.6%) | 1952 (17.8%) | 17795 (17.8%) |  |
| East South Central | 1668 (5.3%) | 2632 (4.6%) | 563 (5.1%) | 4863 (4.9%) |  |
| West North Central | 2492 (7.9%) | 4774 (8.3%) | 896 (8.2%) | 8162 (8.2%) |  |
| West South Central | 1588 (5.0%) | 2735 (4.8%) | 626 (5.7%) | 4949 (4.9%) |  |
| Mountain | 1388 (4.4%) | 3057 (5.3%) | 507 (4.6%) | 4952 (4.9%) |  |
| Pacific | 2848 (9.0%) | 5379 (9.4%) | 1073 (9.8%) | 9300 (9.3%) |  |
| Year of Diagnosis |  |  |  |  | <0.0001 |
| 2010 | 1326 (4.1%) | 2658 (4.5%) | 585 (5.2%) | 4569 (4.5%) |  |
| 2011 | 1915 (6.0%) | 3457 (5.9%) | 712 (6.3%) | 6084 (5.9%) |  |
| 2012 | 2288 (7.1%) | 3843 (6.5%) | 717 (6.3%) | 6848 (6.7%) |  |
| 2013 | 2838 (8.8%) | 4349 (7.4%) | 915 (8.1%) | 8102 (7.9%) |  |
| 2014 | 2861 (8.9%) | 4820 (8.2%) | 1014 (9.0%) | 8695 (8.5%) |  |
| 2015 | 3104 (9.7%) | 5367 (9.1%) | 1115 (9.9%) | 9586 (9.4%) |  |
| 2016 | 3354 (10.4%) | 6062 (10.3%) | 1306 (11.5%) | 10722 (10.5%) |  |
| 2017 | 3648 (11.3%) | 6450 (11.0%) | 1358 (12.0%) | 11456 (11.2%) |  |
| 2018 | 3548 (11.0%) | 6928 (11.8%) | 1224 (10.8%) | 11700 (11.4%) |  |
| 2019 | 3936 (12.2%) | 7825 (13.3%) | 1266 (11.2%) | 13027 (12.7%) |  |
| 2020 | 3340 (10.4%) | 7132 (12.1%) | 1096 (9.7%) | 11568 (11.3%) |  |

Abbreviations: CI = confidence interval; OS = overall survival

**Table S3:** Comparison of low, intermediate, and high Oncotype DX Kaplan-Meier curves within each TNM substage

| **TNM substage** | **Log-rank *p*-values** |
| --- | --- |
| pT1mi pN0 | *p* = 0.691 |
| pT1mi pN+ | **** |
| pT1a pN0 | *p* = 0.025 |
| pT1a pN+ | *p* = 0.737 |
| pT1b pN0 | *p* < 0.001 |
| pT1b pN+ | *p* = 0.002 |

**Could not estimate KM curve due to small numbers.

**Table S4**: 5-year OS by TNM substage and Oncotype DX group

|  | **5-year OS** | | |
| --- | --- | --- | --- |
|  | **Low Oncotype DX** | **Intermediate Oncotype DX** | **High Oncotype DX** |
| **TNM substage** |  |  |  |
| pT1mi pN0 | 92.8% (85.4-96.5%) | 83.5% (65.7-92.6%) | 90.3% (76.1-95.2%) |
| pT1mi pN+ | ** | ** | ** |
| pT1a pN0 | 97.1% (96.5-97.7%) | 97.6% (97.1-98.0%) | 96.7% (95.5-97.6%) |
| pT1a pN+ | 96.1% (92.8-97.9%) | 98.4% (96.5-99.3%) | 97.7% (93.1-99.3%) |
| pT1b pN0 | 97.0% (96.7-97.2%) | 97.5% (97.3-97.6%) | 96.0% (95.6-95.5%) |
| pT1b pN+ | 97.5% (96.6-98.2%) | 97.6% (96.9-98.1%) | 95.3% (92.7-97.0%) |

**Could not calculate 5 -year OS due to small numbers.

**Table S5**: 5-year OS by TNM substage, age subgroup, and Oncotype DX group

|  | **5-year OS** | | | | | |
| --- | --- | --- | --- | --- | --- | --- |
|  | **Low Oncotype DX** | | **Intermediate Oncotype DX** | | **High Oncotype DX** | |
| **Age subgroup** | <50 | ≥50 | <50 | ≥50 | <50 | ≥50 |
| **TNM substage** |  | | | | | |
| pT1mi pN0 | ** | 91.5% (83.0-95.9%) | ** | ** | 76.6% (43.3-91.9%) | 96.7% (78.8-99.5%) |
| pT1mi pN+ | ** | ** | ** | ** | ** | ** |
| pT1a pN0 | 99.7% (98.7-99.9%) | 96.5% (95.7-97.1%) | 99.6% (98.9-99.9%) | 96.9% (96.3-97.5%) | 98.9% (96.5-99.6%) | 96.1% (94.5-97.2%) |
| pT1a pN+ | 97.1% (88.6-99.3%) | 95.7% (91.6-97.9%) | ** | 97.8% (95.2-99.0%) | ** | 99.6% (89.7-98.9%) |
| pT1b pN0 | 99.4% (99.1-99.6%) | 96.5% (96.2-96.8%) | 99.3% (99.1-99.5%) | 97.0% (96.8-97.2%) | 97.1% (95.9-98.0%) | 95.9% (95.3-96.3%) |
| pT1b pN+ | 98.8% (96.7-99.5%) | 97.2% (96.1-98.0%) | 99.0% (97.8-99.6%) | 97.1% (96.2-97.8%) | 98.4% (93.9-99.6%) | 94.3% (91.0-96.5%) |

**Could not calculate 5 -year OS due to small numbers.

**Supplemental Figures**

**Figure S1**: Unmatched Univariate Comparison of OS By Receipt of Chemotherapy Among Patients with High Genomic Risk pT1mi/a/b pN0-N1mi HR+BC


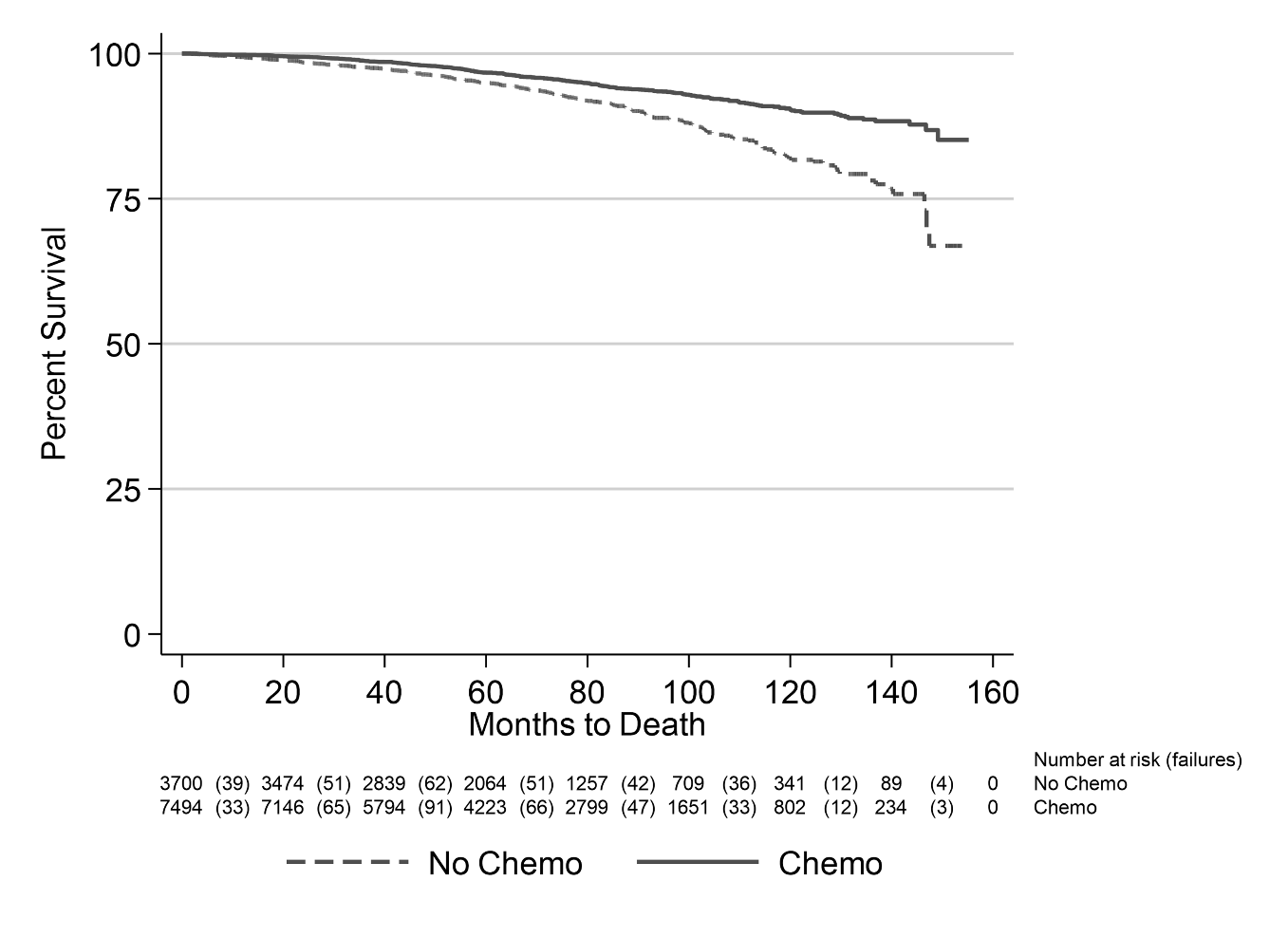


**Figure S2**: Unmatched Univariate Comparison of OS By Receipt of Oncotype Testing Among Patients with pT1mi/a/b pN0-N1mi HR+BC


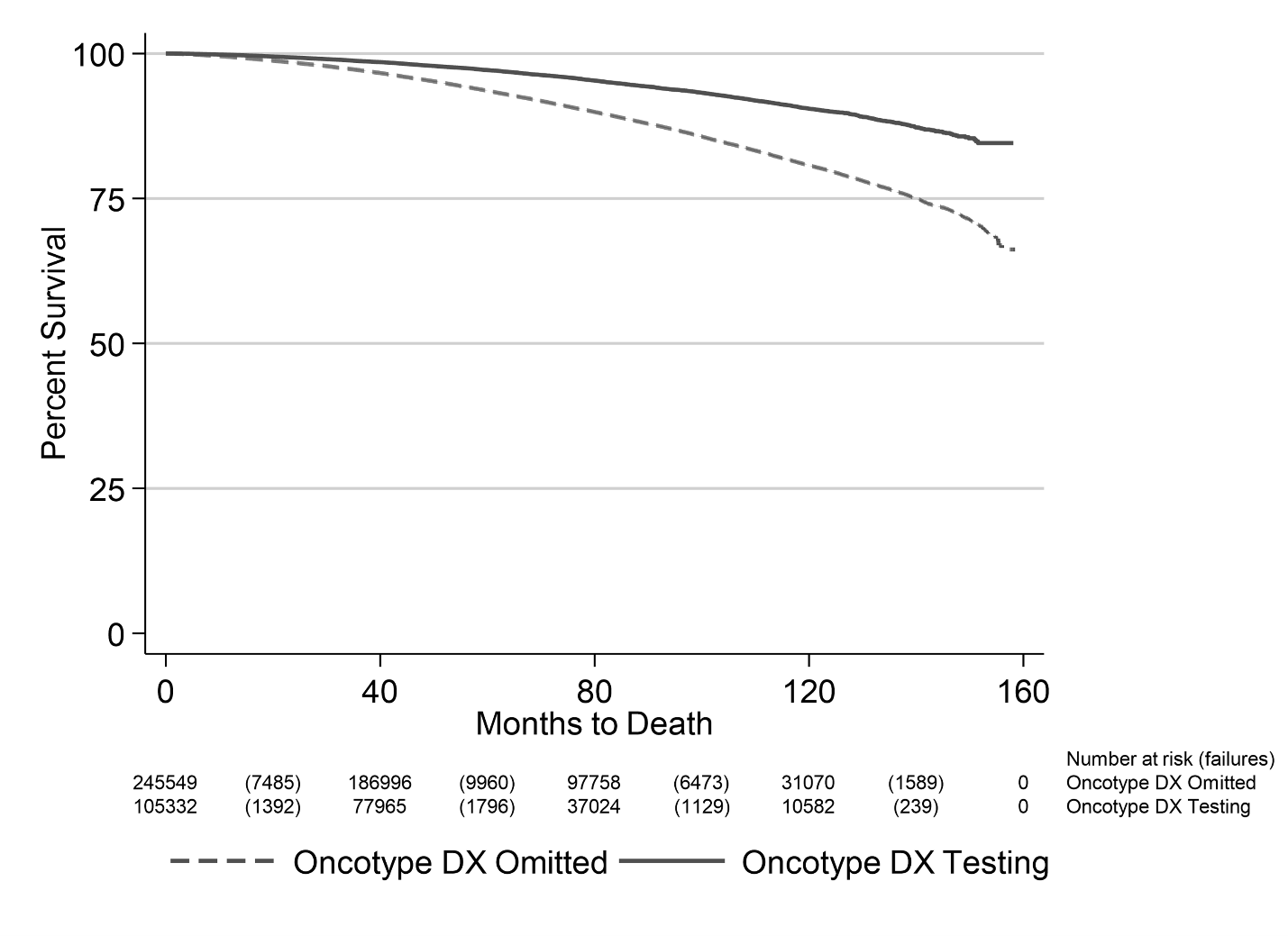


**Figure S3**: Unmatched Univariate Comparison of OS By Oncotype DX Risk Categorization Among Patients with pT1mi/a/b pN0-N1mi HR+BC Independent of Chemotherapy Receipt.


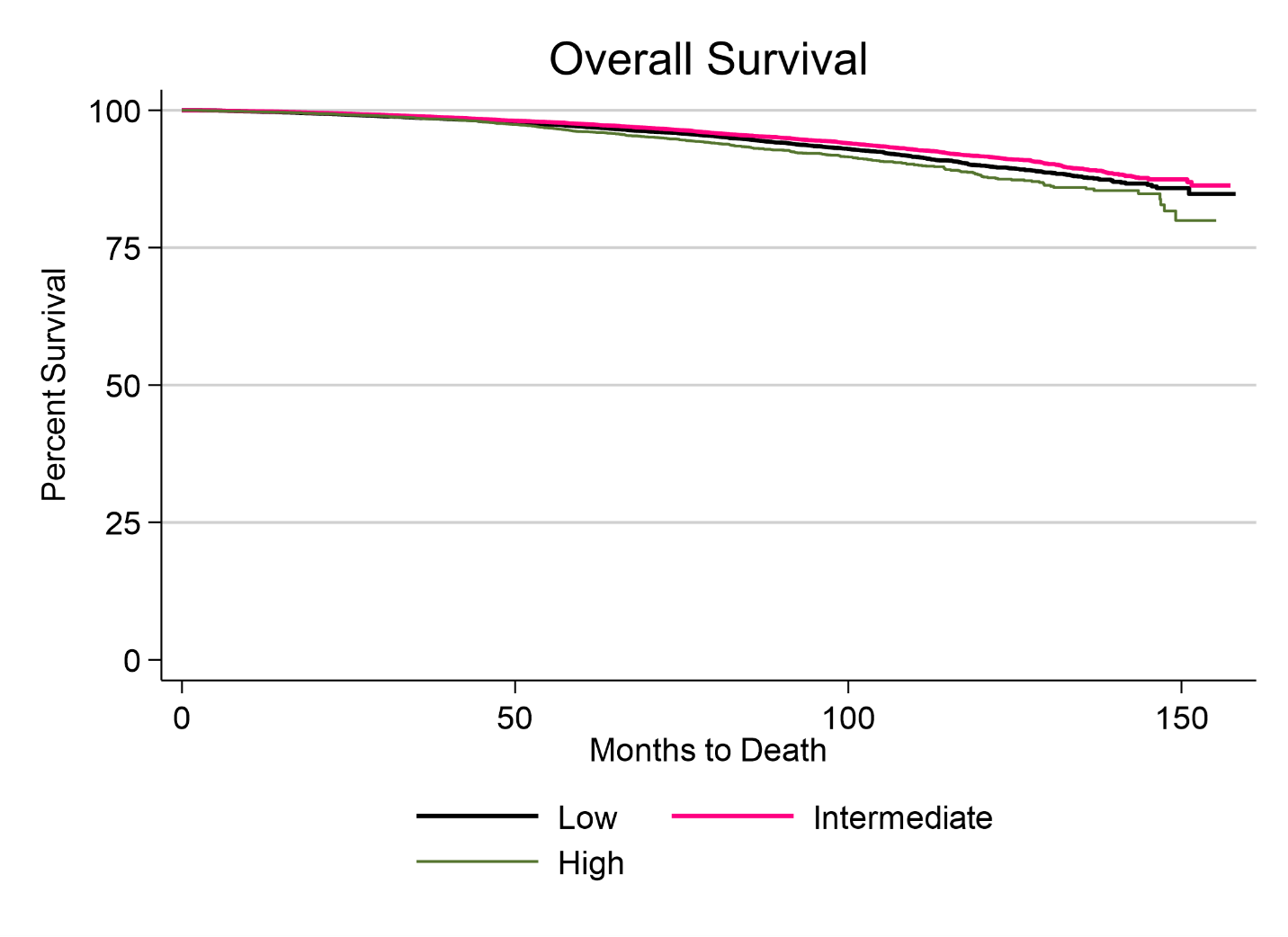


**Figure S4**: Unmatched Univariate Comparison of OS By Both Chemotherapy Receipt & Oncotype DX Risk Categorization Among Patients with pT1mi/a/b pN0-N1mi HR+BC


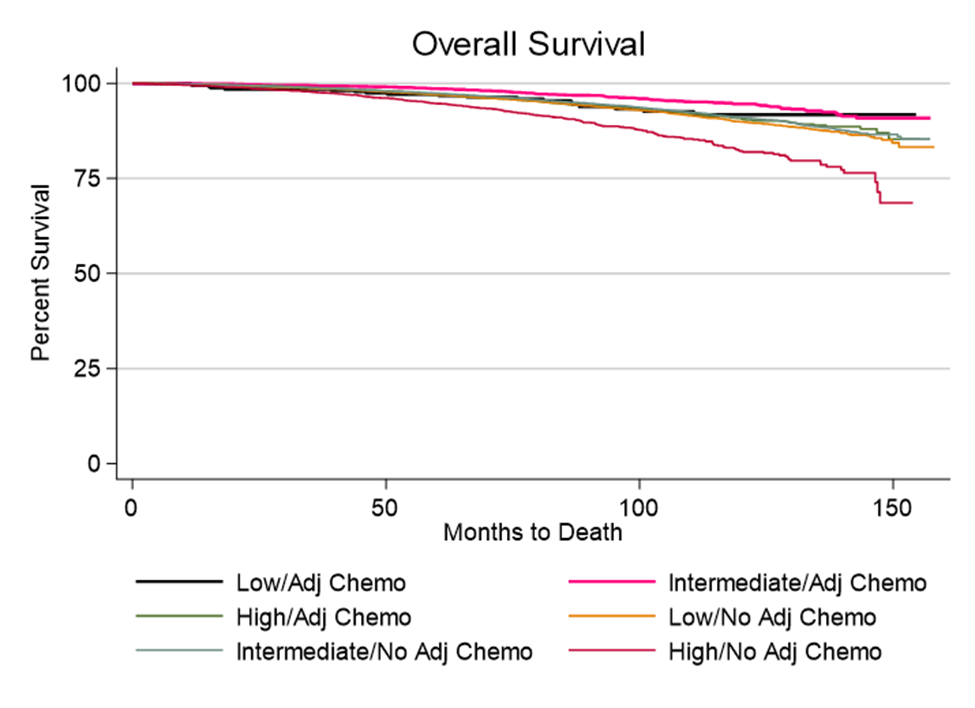


**Figure S5**: Unmatched Univariate Comparison of OS By Both Chemotherapy Receipt & Oncotype DX Risk Categorization Among Patients with pT1mi HR+BC


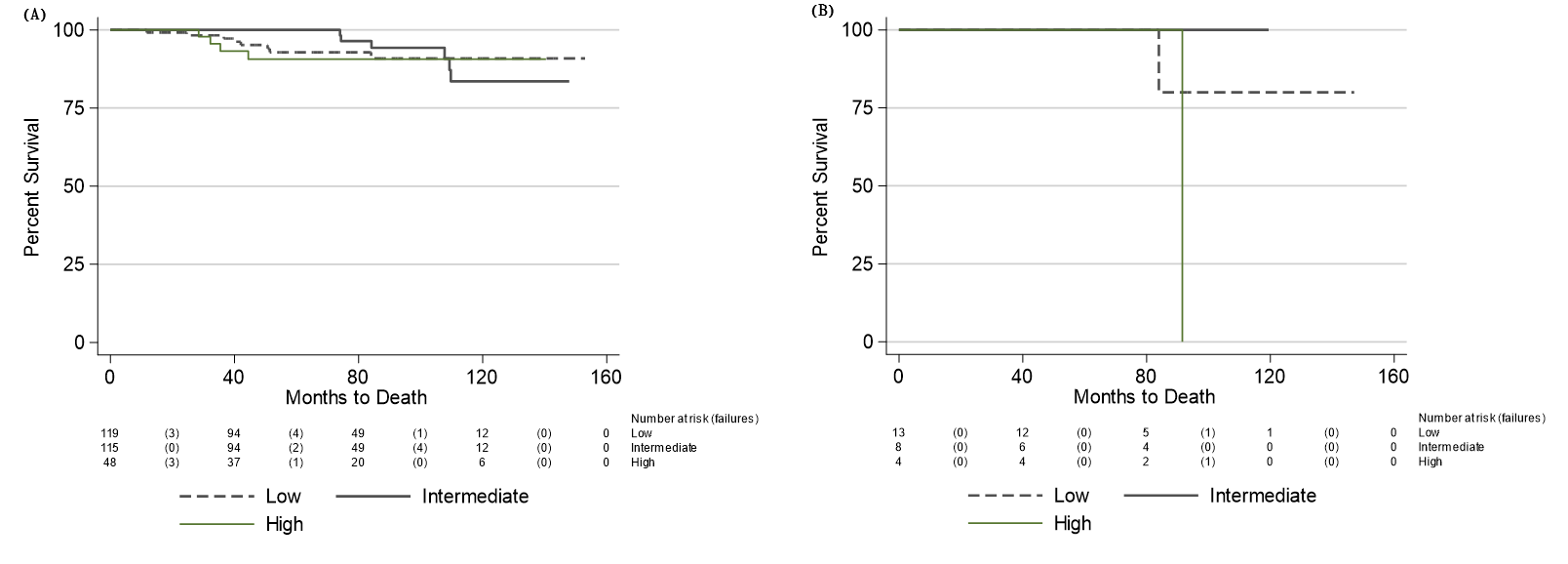


1. OS for pT1mi pN0 HR+BC (B) OS for pT1mi pN+ HR+BC

**Figure S6**: Unmatched Univariate Comparison of OS By Both Chemotherapy Receipt & Oncotype DX Risk Categorization Among Patients with pT1a HR+BC


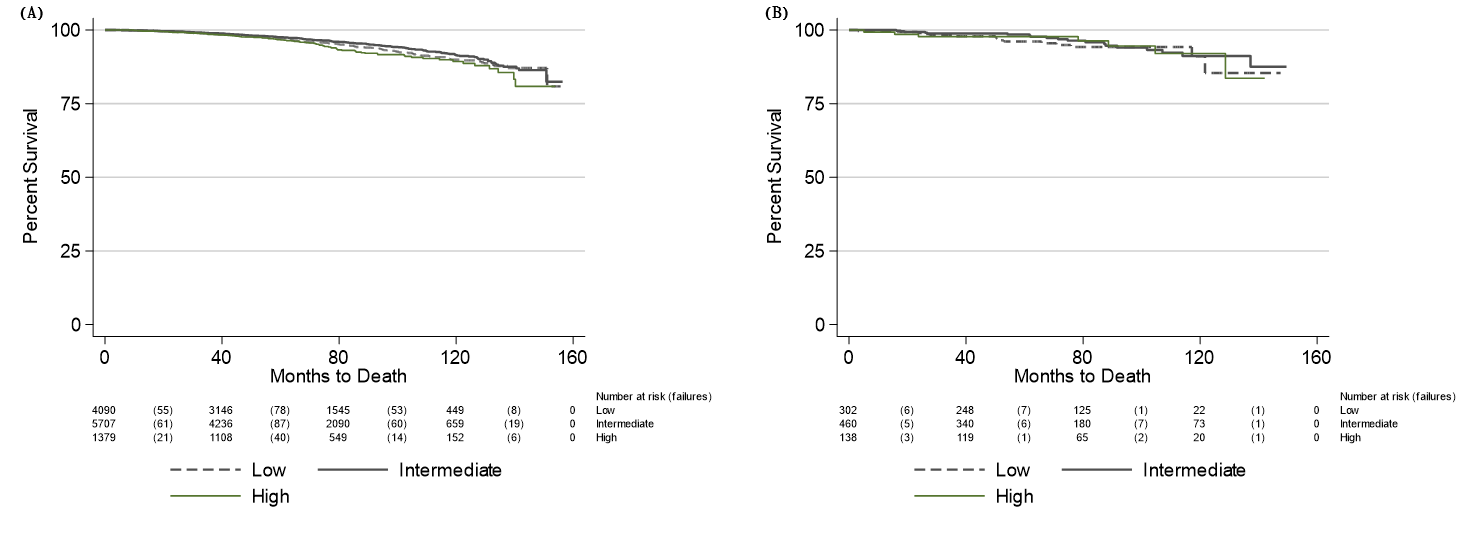


1. OS for pT1a pN0 HR+BC (B) OS for pT1a pN+ HR+BC

**Figure S7**: Unmatched Univariate Comparison of OS By Both Chemotherapy Receipt & Oncotype DX Risk Categorization Among Patients with pT1b HR+BC


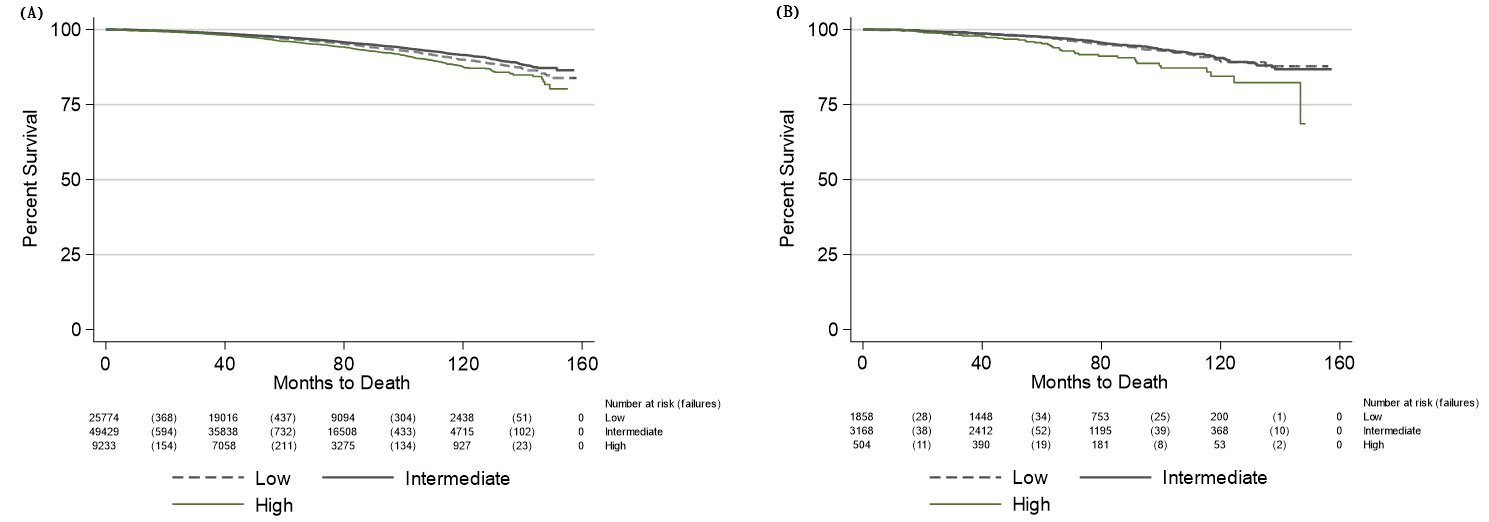


1. OS for pT1b pN0 HR+BC (B) OS for pT1b pN+ HR+BC
